# Patient non adherence to tuberculosis treatment in Sudan: socio demographic factors influencing non adherence to tuberculosis therapy in Khartoum State

**DOI:** 10.11604/pamj.2016.25.80.9447

**Published:** 2016-10-17

**Authors:** Ahmed Osman Ahmed Ali, Martinus Hendrik Prins

**Affiliations:** 1 Ministry of Health, Saudi Arabia, Maastricht University Medical Centre, Maastricht, the Netherlands; 2 Maastricht University Medical Centre, Maastricht, the Netherlands

**Keywords:** Tuberculosis, non-adherence, adherence, defaulter, compliance

## Abstract

**Introduction:**

Despite the Treatment pulmonary TB patients, defaulting from treatment may remain the major challenge to control TB. In addition, it increases the risk of drug resistance, relapse, and death and may prolong infectiousness. Our objective was to identify determinants of treatment defaulting among TB patients in Khartoum State, Sudan.

**Methods:**

We conducted a case-control study where the patients defaulting from treatment were considered as ‘cases’ and those completing treatment as ‘controls’. Between May 2010 to May 2011.

**Results:**

There were 2727 TB patients who attended TB treatment clinics during study period. Out of these 2399 patients (86%) had continued their treatment while 328 patients (14%) had interrupted it. 105 cases were traced and interviewed. In addition 210 patients who had continued their treatment were included (controls). In the multivariate analysis the variables that remained in the model were: residential locality (rural area) (OR 2.58; 95% CI 1.4 -4.67), patients moving or changing address (OR 5.47; 95% CI 2,90- 10-35), absence of family support (OR 2.14; 95% CI 1.12 - 4.11), and occupation (blue collar work) (OR 2.38; 95% CI 1.39 -4.10).

**Conclusion:**

The results of this study conclude some socio-demographic factors influence defaulting of TB treatment. We believe that the findings are applicable to current situation of TB management and control in Sudan and other developing countries

## Introduction

Tuberculosis (TB) as a disease has been known ever since the dawn of man's history [1]. In 1993 the World Health Organization (WHO) declared that TB was the major global public health problem [2]. It is estimated that one-third of the world's population (approximately two billion people) have been affected by the mycobacterium tuberculosis [3–5]. The WHO estimates that currently about 9.4 million new TB cases occur each year and that approximately 1.8 million deaths annually are related to TB [6, 7]. In 2010, it was estimated that in Sudan there were 209 cases of active TB per 100.000 of populace with an annual incidence of new cases of 119/100.000, resulting in approximately 37.000 new cases each year in Sudan. Hence, Sudan shoulders about 15% of TB burden in the Eastern Mediterranean Region and has the second highest active TB prevalence of the countries in this region. In addition, the estimated death rate related to TB, including HIV infected TB patients, was 24/100.000 per year [8]. Treatment of active pulmonary TB patients remains the most effective strategy to stop the spread of the disease [9, 10]. Defaulting from treatment may remain the major challenge to control TB. In addition, it increases the risk of drug resistance, relapse, and death and may prolong infectiousness [11–15]. Non-compliance with therapy is considered a priority for researchers because it remains unclear how to identify patients at risk for non-compliance, or how to effectively intervene with such patients [16]. The high rate of patient defaulting TB treatment in Khartoum State makes the identification of risk factors leading to this default essential. The present study was conducted to identify determinants of treatment defaulting among TB patients. Such information could help to put forward suggestions and recommendations that can lead to reduction of TB treatment defaulting.

## Methods

This was an observational case control study where the patients defaulting from treatment were considered as ‘cases’ and those completing treatment as ‘controls’.

**Setting:** This study was conducted in Khartoum State. In 1993, the Ministry of Health in Khartoum State established a tuberculosis control program. The decentralized healthcare system in Khartoum is divided into seven districts and 19 health areas. Its health facilities include 43 hospitals, 147 health centers, 185 NGOs centers, 235 dispensaries and 365 primary health care units. TB services are delivered in primary health care along with all other routine health services. A registered nurse is designated responsible for treatment and follow up for continuation of treatment in the primary health care unit. This primary health care unit is the basic unit of management of the program and also the unit of reporting. Personnel at the primary health care unit responsible for tuberculosis services include a medical assistant, a laboratory technician and a clerk. The program provides care through the DOTS strategy (Directly Observed Treatment with Short course chemotherapy) as recommended by WHO. TB patients receive their treatment through 53 TB treatment units distributed all over the state [17].

**Population:** The reference population for this study comprised all tuberculosis patients registered at tuberculosis centers at all provinces in Khartoum state from May 2010 to May 2011. The data collection was done from 1th of May 2011 to 15th of July 2011. The inclusion criteria for both cases and controls were; patients age more than 15 years and clinically and laboratory diagnosed as tuberculosis, registered at the treatment units in Khartoum States. Cases were those patients identified as TB treatment defaulting during the data collection period. Following identification of each case (defaulter) without exclusion criteria, the next 2 subsequent patients without exclusion criteria, who came for treatment or follow up, without defaulting, in the same TB treatment unit or the near one in the same area, were taken as control into the study. The patients were excluded from the study if they were: too ill for interview, had a psychiatric illness, or gave incorrect address and could not be traced. The following definitions were applied according to the World Health Organization (WHO, 2002). Treatment default: an interruption of TB treatment for two or more consecutive months during the intended treatment period. Pulmonary TB: a patient with tuberculosis disease involving the lung parenchyma. Extra-pulmonary TB: a patient with tuberculosis of organs other than the lungs (e.g. pleura, lymph nodes, abdomen, genitourinary tract, skin, joints and bones, meninges). Diagnosis should be based on a culture-positive specimen, histological evidence or strong clinical evidence consistent with active extra-pulmonary disease, followed by a decision by a clinician to treat with a full course of anti-tuberculosis chemotherapy. A patient in whom both pulmonary and extra-pulmonary TB has been diagnosed was classified as pulmonary TB [18–20].

**Data Collection:** Information on disease related factors and treatment related factors were retrieved from patients´ medical records. In addition, a face to face interview was held, using a standardized questionnaire by trained interviewers to elicited information on the various factors possibly associated with treatment defaulting. The following variables were collected: socio-demographic factors including; age, sex, ethnicity, marital status, educational level, occupation, employment status, family income, nationality, residential locality, distance of residence from treatment center, religion, patients moving or changing address, family size, house size, means of transport to the health center, travelling cost to health centre, waiting time, family support and site of tuberculosis [9, 11, 21–28]. Before the start of data collection the interviewers had been trained on how to interview the respondents, and had been given instructions on how to fill the questionnaire. After that, pretesting was conducted by interviewing few patients. Based on the pretesting results the questionnaire was used without any major changes.

**Statistics:** The sample size was calculated according to Fleiss J.L(1981) [29] using a two sided type one error of 0.05 and a power of 80% and the ability to detect an odds ratio of 2.0 with a exposure frequency of 30% in the control group and a ratio of cases to controls of 1:2. This yielded a sample size of 105cases and 210 controls. Data were reviewed for consistency and completeness. Data analysis was performed in SPSS (Statistical package of Social Sciences) version 16. The Demographic characteristics of cases and controls were compared using test for qualitative variables and student's t tests for continuous variables. Univariate and multivariate analysis were conducted. Descriptive statistics were calculated for all dependent variables. Logistic regression was used to calculate the odds ratio and its 95% confidence interval. Variables that were related to treatment default with a p-value less than 0.20 were entered in a multivariate model, using a backward approach [30, 31].

**Ethical Considerations:** Ethical approval was obtained from Ministry of Health Khartoum State ethical Committee. Permission was granted by public committee leaders in the localities through official letters. Informed verbal consent was secured from every eligible patient included in this study before the interview. Privacy and confidentially was maintained. Prior to the arrival of the data collection team the respondents had been informed regarding all relevant aspects of the study, including the purpose of the study, interview process and potential benefits. The interviewers introduced themselves to respondents and outlined the scope of interview and its approximate length to the potential respondents at the beginning of each interview. The respondents had been informed that the participation was entirely voluntary, and that privacy and confidentially will be maintained during data processing and reporting. Potential respondents also were informed that they had the right to refuse to participate, or to end the interview at any time.

## Results

**Patients:** There were 2727 TB patients who attended TB treatment clinics during study period. Out of these 2399 patients (86%) had continued their treatment while 328 patients (14%) had interrupted it. Out of these, 185 patients had defaulted prior to the data collection period. Hence, 143 patients were potentially eligible as cases. Of these 15 had given a wrong address and 12 had moved out of Khartoum State and could not be interviewed. A further 11patients refused the interview. Hence, 105 cases were traced and interviewed. In addition 210 patients who had continued their treatment were included (controls). The demographic and TB characteristics are given in (Table 1, Table 2). Cases and controls were of similar age, but cases lived more often in a village and at a greater distance from the TB center. Also, they were more often illiterate, had less family support and were more liable to give a wrong address or to move during treatment period without informing the treatment center.

**Table 1:** Demographic and social characteristics of the study population

Socio-demographic factors	Cases N=105	Control N=210	P-value
Age-mean (SD*)	32.8 (14.4)	34.6 (14.9)	0.339
Between 15 and 30 years	56 (53.3%)	100 (47.6%)	
Over 30 years	49 (46.7%)	110 (52.4)	
**Sex**			0.98
Male	74 (70.5%)	128 (60.9%)	
Female	31 (29.5%)	82 (30.1%)	
**Site of tuberculosis**			0.39
Pulmonary	92 (87.6%)	180 (85.7%)	
Extra-pulmonary	13 (12.4%)	30 (14.3%)	
**Type of residential area**			0.001
City	65 (61.0%)	169 (80.5%)	
Village	41 (39.0%)	41 (19.5%)	
**Distance to clinic**			0.005
Between 1 and 5 kilometers	35 (33.3%)	105 (50.0%)	
More than 5 kilometers	70 (66.7%)	105 (50.0%)	
**Patient moving or giving wrong address**			0.001
Yes	41 (39.0%)	23 (11.0%)	
No	64 (61.0%)	187 (89.0%)	
**Inform clinic when moving**			0.002
Yes	7(17.1%)	10 (43.5%)	
No	34 (82.9%)	13 (56.5%)	
**Type of transport to get to clinic**			0.103
On foot or by bicycle	7(6.7%)	25(11.9%)	
With car or public transport	98(93.3%)	185(88.9%)	
**Transportation cost**			0.06
Less than 3 Sudanese pound(SD)	61(58.1%)	101(48.1%)	
3 Sudanese pound(SD) or more	44(41.9%)	109(51.9%)	
**Time to clinic**			0.048
Up to 60minutes	82(79%)	184(87.6%)	
More than 60minutes	22(21%)	26(22.4%)	

**Table 2:** Demographic and social characteristics of the study population ( part 2)

Socio-demographic factors	Cases(Non-adherence) 105 (33, 3%)	Control(adherence) 210 (66.7%)	P-value
**Marital status**			0.178
Single	50 (47.6%)	87 (41.4%)	
Married	55 (51.4)	123 (58.6)	
**Family size**			0.484
Less than 4 members	17(16.2%)	36 (17.1%)	
More than 4 members	88(83.8%)	174 (82.9%)	
**House size(room number)**			1.00
Less than 3rooms	41 (39%)	82(39%)	
More than 3 rooms	64(69%)	128(61%)	
**Family income =(mean, SD)**	(1.14, 0.447 )	(1.24, 0.575 )	0.07
Less than 1000 Sudanese pound(SP)	93(88.6%)	171(81.4%)	
More than 1000(SP)	12(11.4%)	39 (18.6%)	
**Family support**			0.001
Those with family support	73(69.5%)	180(85.7%)	
Those without family support	32(30.5%)	30(14.3%)	
**Occupation**			0.001
Blue collar work	66(62.9%)	91(43.3%)	
White work	39(39.1%)	119(52.7%)	
**Nationality**			0.523
Sudanese	98(93.3%)	197(93.8%)	
Non-Sudanese	7(6.7%)	13 (6.2%)	
**Educational level**			0.005
Illiterate	24 (22.9%)	23(11%)	
Literate	81 (77.1%)	187(89%)	
**Religion**			0.370
Muslim	95(90.1%)	196(92.3%)	
Non muslim	10(9.9%)	14(7.7%)	

**Risk factors for defaulting:** in the univariate analysis the socio demographic factors found statistically significant (p<0.05) related to TB patient treatment default were: educational level (illiterate), distance to health center, residential locality (rural area), patients moving or changing address, time to clinic >60minutes, no family support and, occupation (blue collar work) Table 3. In the multivariate analysis the variables that remained in the model were: residential locality (rural area), patients moving or changing address, absence of family support and occupation (blue collar work). The adjusted odds ratios with their corresponding 95% confidence intervals are given in Table 4.

**Table 3:** Socio-demographic factors associated with TB treatment default

Socio-demographic factor	Odds ratio (OR)	95% C.I
Age group		
Between 15 and 30 years vs. Over 30 years	0.80	0.50 -1.27
Sex		
Male vs. Female	1.53	0.93-2.53
Site of tuberculosis		
Pulmonary vs. Extra-pulmonary	1.18	0.60-2.37
Residential locality		
Village vs. City	2.64	1.57-4.44
Distance		
More than 5 kilometers vs Between 1 and 5 kilometers	2.000	1.23-3.26
Patient movement		
Patient moving or giving wrong address vs. Those not moved	5.21	2.90-9.34
Inform clinic when moving		
Those not informed the clinic vs. Those Inform clinic when moving	6.31	1.98- 20.11
Type of transport to get to clinic		
On foot or by bicycle vs With car or public transport	1.89	0.80-4.53
Transportation cost		
Cheap vs Expensive	0.67	0.42- 1.10
Time to clinic		
More than 60minutes vs Up to 60 minutes	1.88	1.01-3.50
Marital status		
Single vs Married	1.29	0.80-2.06
Family size		
Less than 4 member vs More than 4 members	0.93	0.50 -1.76
House size(room number(		
Less than 3rooms vs 3 rooms or more	1.00	0.62- 1.62
Family income		
Less than1000 Sudanese pound(SP) vs 1000(SP) or more	1.77	0.88- 3.54
Family support		
Those without family support vs. Those with family support	2.63	1.49-4.64
Occupation		
Blue collar work vs White work	2.21	1.37- 3.58
Nationality		
Non-Sudanese vs. Sudanese	1.08	0.42- 2.80
Educational level		
Illiterate vs Literate	2.64	1.57-4.44
Religion		
Muslim vs Non muslim	0.70	0.29-1.55

**Table 4:** Adjusted Odds Ratio (the model) factors associated with non- adherence (OR and 95% C,I)

Socio-demographic factors	Odds ratio (OR)	95% C.I
**Residence**		1.43 - 4.67
(rural versus urban)	2.58	
**Occupation**		1.39 -4.10
(blue collar work vs white work)	2.38	
**Family support**		1.12 - 4.11
those without family support vs those with family support	2.14	
**Patient movement**		2,90- 10-35
those moving or giving wrong address vs those not moved during treatment period	5.47	

## Discussion

The results of present study showed that 14% of TB patients in Khartoum State were treatment defaulters. Also we identified several factors associated with TB treatment default which included: educational level (illiterate), distance to health center (more than 5 kilometer), type of residential area (village), moving, absence of family support, and occupation (blue collar work). Surprisingly, the traditional factors thought to be related to TB treatment default (e.g. age, religion, family income, family size, house size and travelling cost) were found not statistically associated with default in this study. The findings of this study are similar to results in developing countries with low resources in Africa and Asia which carry the highest burden of TB [22–28]. We observed that patients moving or giving wrong address were more likely to default their TB treatment. These findings are similar to results of other studies in developing (Uganda, South Africa) and developed countries (USA) [21, 26, 28]. However, in a recent study in Malaysia, changing of residence was not associated with TB default [22]. We could confirm that rural residence, distance to health center (more than five kilometers), educational level (illiteracy), and absence of family support were strongly associated with TB default. Hence the influence of these factors on defaulting TB treatment is well recognized as mentioned by Tatek in (Ethiopia), [23] Bernard N Muture in (Kenya) [9] and Samuel A (Ghana) [25]. However our findings were in contrast to those reported by Nyi and Chuah from Malysia the rural residence, distance to health center, and educational level were not associated with TB treatment default [11, 22]. The present study showed no significant association between TB treatment default and the following socio-demographic factors: age, sex, ethnicity, marital status, nationality, means of transport to the health center, waiting time, religion, family income, family size, house size, travelling cost the and site of tuberculosis. These findings are similar to those reported by Nyi from Malaysia, but differ from those reported by Jaggarajamma K. in India, Connoly C. in South Africa and Kelly E Dooley in Morocco where age (older more than 45 years/younger less than 45 years) and female sex were related to TB treatment default.

The results of this study might help the policy maker in Khartoum state, Sudan and developing countries in planning and policy development to strengthen TB control programs in general. Attention for and exploring of the factors that are -even with the DOTS-approach-still strongly associated with TB treatment default could be done. An important issue seems health education and counseling provided by health workers for TB patients including their families. These efforts should encourage patients and their families to adhere to TB treatment and also help to ensure family support during the treatment period. Changing address and moving to other place continues to be a challenge for TB treatment adherence. TB patients and their families should give their home addresses and be informed to notify the health personnel´s if they move. Possibly, recording of the address of a (more distant) family member living at a different location could be used to increase tracking ability of the health care workers. This study showed that those who lived in the rural areas or if the distance of their residences is more than five kilometers from the TB treatment centre were more than two times likely of being non-compliance to TB treatment than those lived in urban areas or their residence is less than 5 kilometers to TB treatment centers. Hence, the Provision of health services and accessibility in the rural areas and near to residence of TB patient will enhance the patients’ compliance to treatment. For those who had no work or of low socioeconomic status. They need more attention from the governmental and nongovernmental organizations to support TB patients financially and socially. Overall the results of present study raise very important issues on TB default and socio-demographic predictors’ factors in Sudan since there were no previous details studies on this field. This results can help in decreasing the TB treatment default. Hence, decreasing treatment failure, multi drugs resistant, treatment relapse and spreading of tuberculosis in the community.

There are some methodological aspects of this study need attention: firstly, this study was conducted in Khartoum State, capital of Sudan which is the most populated state in Sudan. The population in this state could be safely stated to represent the whole country as most of the inhabitants come from various parts of Sudan. Hence, the results of this study apply to other parts of Sudan. In addition, the TB patients included in this study were selected from all tuberculosis treatment units (health centers and hospitals) in the state. By this fact, the generalization of the study findings to total tuberculosis population in the state and Sudan could be done and seems logical. Secondly, the recall bias was minimized by reviewing the patient medical records and cross checking for each study variables, and avoiding rush questioning during interview period. Thirdly, the reliability and sensitivity of information gathered from each subject could not be counterchecked. Although questions about sensitive issues were carefully tackled using a warm approach and ensuring strict and uninterrupted communications, so as to maximize the validity of the responses obtained. Fourthly, possible confounders were taken into consideration in the design (by restricting the diagnosis criteria) and by using logistic regression. Lastly, the major problem we faced during this study was how to reach the defaulting patients (cases) for this study. This problem was tackled stepwise. Firstly, their medical records were traced and identified and all contact information was reviewed. Then, study personnel used the following sequence of contact attempts: calls-first to the patient and thereafter to known family members or friends - and home visits - first to patient and thereafter to known family members or friends. Interestingly, it appeared during the study that many of the defaulting patients did not have access to mobile telephones, a risk factor not previously described. The interviewers made an average of three attempts to contact each defaulter before deciding that a defaulter was a non-respondent. Due to the proportion of defaulters who could not be traced and retrieved, the generalizability of the findings to the whole population of patients with tuberculosis should be done with caution.

## Conclusion

The results of this study conclude some socio-demographic factors (rural residence, occupation (blue colour work), those without family support and those moving or change their address during treatment period) influence defaulting of TB treatment. We believe that the findings are applicable to current situation of TB management and control in Sudan and other developing countries.

### What is known about this topic

- TB is a major health problem in Sudan;
- defaulting from treatment remain the major challenge to control TB disease;
- Defaulting from treatment increases the risk of drug resistance, relapse, and death and may prolong infectiousness.

### What this study adds

- This study provides valuable information on risk factors leading to TB treatment default in Sudan;
- The current study confirms the high default rate in Khartoum state which mentioned in previous studies;
- Special attention should be given to TB patients and their families and patients address to enhance treatment compliance.
